# Effects of conventional versus multimodal vestibular rehabilitation on functional capacity and balance control in older people with chronic dizziness from vestibular disorders: design of a randomized clinical trial

**DOI:** 10.1186/1745-6215-13-246

**Published:** 2012-12-31

**Authors:** Natalia Aquaroni Ricci, Mayra Cristina Aratani, Heloisa Helena Caovilla, Fernando Freitas Ganança

**Affiliations:** 1 Department of Otorhinolaryngology and Head & Neck Surgery, Federal University of São Paulo, Otoneurology Discipline, Rua Pedro de Toledo 947, Vila Clementino, São Paulo 04025-002, Brazil

**Keywords:** Dizziness, Rehabilitation, Vestibular diseases, Aged, Postural balance, Disability evaluation

## Abstract

**Background:**

There are several protocols designed to treat vestibular disorders that focus on habituation, substitution, adaptation, and compensation exercises. However, protocols that contemplate not only vestibular stimulation but also other components that are essential to the body balance control in older people are rare. This study aims to compare the effectiveness of two vestibular rehabilitation protocols (conventional versus multimodal) on the functional capacity and body balance control of older people with chronic dizziness due to vestibular disorders.

**Methods/design:**

A randomized, single-blind, controlled clinical trial with a 3 months follow-up period will be performed. The sample will be composed of older individuals with a clinical diagnosis of chronic dizziness resulting from vestibular disorders. The subjects will be evaluated at baseline, post-treatment and follow-up. Primary outcomes will be determined in accordance with the Dizziness Handicap Inventory (functional capacity) and the Dynamic Gait Index (body balance). Secondary outcomes include dizziness features, functional records, body balance control tests, and psychological information. The older individuals (minimum sample *n* = 68) will be randomized to either the conventional or multimodal Cawthorne&Cooksey protocols. The protocols will be performed during individual 50-minute sessions, twice a week, for 2 months (a total of 16 sessions). The outcomes of both protocols will be compared according to the intention-to-treat analysis.

**Discussion:**

Vestibular rehabilitation through the Cawthorne&Cooksey protocol has already proved to be effective. However, the addition of other components related to body balance control has been proposed to improve the rehabilitation of older people with chronic dizziness from vestibular disorders.

**Trial registration:**

ACTRN12610000018011

## Background

Aging is characterized by gradual physiological modifications in the body’s systems, which at an advanced age lead to decline of their functions and increased susceptibility to diseases or health disturbances [1]. Among the various consequences of ageing, dizziness requires special professional attention, since it is related to several etiological factors [2]. Furthermore, this symptom is highly prevalent at an advanced age [3], correlates with body unsteadiness and falls [4,5], and negatively impacts daily activities [5] and quality of life [5,6].

Although the dizziness can be attributed to many causes, nearly one-half of these cases are the result of vestibular dysfunction [6-8]. Vestibular dysfunction is present in 18.5% of adults aged 40 to 49 years, in 49.4% of older people aged 60 to 69 years, and up to 84.8% in older people aged 80 years and older [9]. Besides being relatively more prevalent at an advanced age, dizziness could be considered even more dangerous in older people [10], when impairments in other body balance control systems can occur simultaneously [11].

Vestibular dysfunction is typically characterized by vertigo (a sensation of rotatory motion) or body imbalance (disturbances in gaze and postural stability) [9,12]. These symptoms are usually triggered during activities that require head movements, transfers, and ambulation. Consequently, vestibular disorders are frequently reported to cause significant discomfort, to reduce independence during daily activities, and to disturb body balance [9,13].

There are several protocols designed to treat vestibular disorders that focus on habituation, substitution, adaptation, and compensation exercises, recognized as vestibular rehabilitation (VR). In a previous systematic review of the effects of VR on adults and older people with dizziness, it was observed that the Cawthorne&Cooksey protocol was the most common therapeutic approach reported by the selected studies [14].

In a clinical trial, when older individuals were submitted to VR, the Cawthorne&Cooksey protocol was considered effective at controlling body imbalance in all cases, while the Tusa and Herdman protocol was shown to be effective in 87.5% of cases [15]. Both protocols reduced disability in the activities of daily living. In another trial, which also submitted older people with body unsteadiness, vertigo or dizziness (*n* = 215) to the Cawthorne&Cooksey protocol, complete improvement in the vestibular symptoms was observed in 19.3% of the sample [16].

Even though the Cawthorne&Cooksey protocol presents favorable results [15,16], it still lacks exercises for simultaneous management of the proprioceptive and visual information, modification in the base of support, and other motor components. Other studies with multiple-component rehabilitation protocols (balance, flexibility, and strength exercises) have revealed positive results concerning body balance control and the functional capacity of older people [17,18].

According to the evidence that body balance control in older people depends not only on the vestibular system, but also on correlations among all the other systems, it has been proposed that the addition of multiple-component exercises might improve Cawthorne&Cooksey protocol effectiveness. There is also reason to believe that a multimodal protocol might increase older people’s independence in daily activities and body balance control, and as a consequence may reduce falls. Moreover, only a few clinical trials have so far investigated the effects of VR on samples composed exclusively of older individuals; and original and better designed trials are still required in order to provide reliable conclusions [14].

### Aim of the proposed study

This study aims to evaluate and compare the effectiveness of two VR protocols (conventional Cawthorne&Cooksey versus multimodal Cawthorne&Cooksey) concerning functional capacity and body balance control in older people with chronic dizziness derived from vestibular disorders. Secondarily, this clinical trial intends to report sample withdrawals, adverse events and session-to-session VR progress.

## Methods/design

**Figure 1:**
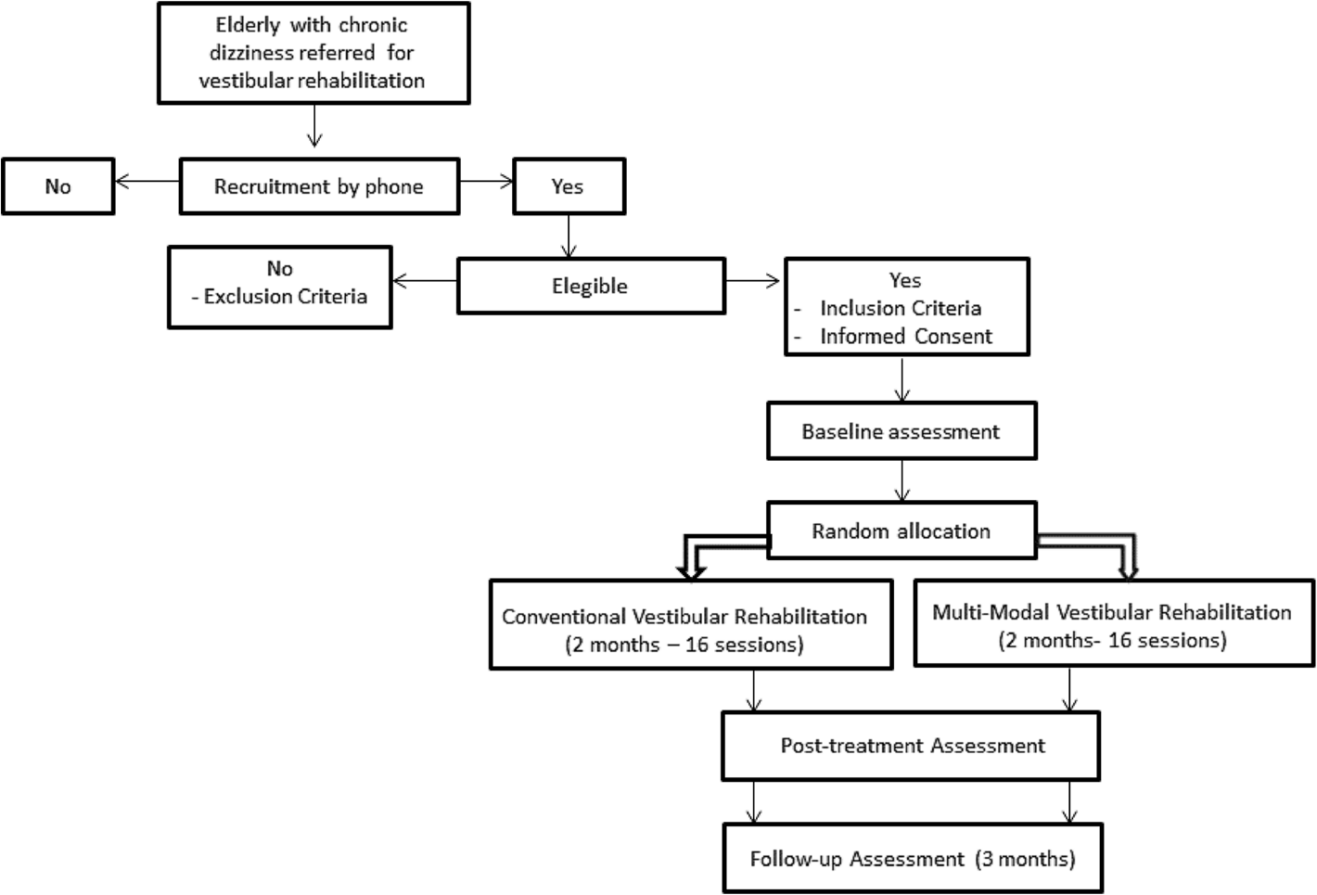
Trial flowchart.

This is a randomized, single-blind, controlled clinical trial with a 3 months follow-up period. The study was approved by the Federal University of São Paulo (UNIFESP) Ethics Review Board (1656/09), and registered in the Australian New Zealand Clinical Trials Registry (ACTRN 12610000018011). The research is reported according to the items stated in the Consolidated Standards of Reporting Trials [19]. Figure 1 shows the flowchart for the research.

### Sample and setting

The sample will be composed of older individuals with complaints of chronic dizziness resulting from vestibular disorders, indicated by the failure to compensate dizziness 2 months or more after the first occurrence [20].

The subjects will be referred to the UNIFESP Otoneurology outpatient clinic, which is an interdisciplinary (otolaryngologists, speech therapists, and physiotherapists) treatment center located in the city of São Paulo, Brazil. The Otoneurology outpatient clinic is part of a public health structure, where chronic dizziness complainers are routinely submitted to anamnesis, otolaryngological examination, and hearing and vestibular tests in order to define the dizziness etiology. Following referral and evaluation, those who require VR will have their names included in an official waiting list for subsequent treatment.

### Recruitment

The patients included in the waiting list will receive a telephone call, during which general information regarding VR will be provided, and they will be asked to take part in the research. For those interested in participating, an appointment will be scheduled at the Otoneurology outpatient clinic to determine their eligibility. Those who are eligible will be asked to sign the informed consent and will be submitted to the baseline evaluation. Patients who do not conform to the inclusion criteria or who refused to take part in the study will be referred for rehabilitation outside the research protocol.

### Inclusion and exclusion criteria

The inclusion criteria are: age 65 years old and over, both genders, and clinical diagnosis of chronic dizziness resulting from a vestibular disorder.

Individuals will be excluded in cases of dizziness not resulting from a vestibular disorder, cognitive deficit (reference values according to the Mini-Mental State Examination considering education level [21]), locomotion requiring a walker or wheelchair, practicing regular physical activities [22], those who were submitted to VR in the previous 6 months, and those under medication for vestibular disorders. Patients presenting benign paroxystic positional vertigo will be also excluded, since the research protocol does not include repositioning maneuvers.

The occurrence of other chronic and disabling diseases will not be considered an exclusion criterion, in order to obtain a representative sample with proper external validity.

### Randomization

Older individuals who meet the inclusion criteria following the baseline assessment and sign the informed consent will be randomized into one of two VR protocols: conventional or multimodal. Randomization will be accomplished by a statistical computer program, by blocks. The block randomization will be gradually performed during the study according to the number of eligible participants registered on the waiting list. Hence, the block sizes will not be fixed and will vary from 4 to 12 subjects, concerning the minimum and maximum capacity for VR at the Otoneurology outpatient clinic. Blocks will therefore eventually be composed of an odd number of participants, and as a consequence unequal groups could be formed. Block randomization was preferred in order to prevent time-related influences from disturbing the homogeneity of both groups over the data collection period (2 years and 6 months). Sample randomization and allocation will be performed by a researcher who is not involved in the clinical trial. In order to assure concealed allocation, the treating therapist will be informed by telephone call regarding the allocation of each participant just before their first session.

### Blinding

Since both interventions are physical exercises, it will not be possible to blind the patient nor the therapist during this research. Hence, this study must be categorized as single blind, since only the outcome assessor will be unaware of intervention assignment. Even though the patients will be consciously under VR, they will be blinded in relation to the protocol they will be involved in. To evaluate the success of blinding, both the outcome assessor and the subjects will be asked at follow-up assessment to provide their opinion concerning which treatment protocol each of the participants was allocated to.

### Outcome measures

Older patients will be submitted to baseline assessment (prior to randomization), and will be retested at post-treatment (8 weeks) and follow-up (3 months). The primary outcome measure to evaluate functional capacity is the Dizziness Handicap Inventory (DHI), while body balance control will be assessed by the Dynamic Gait Index (DGI).

The DHI is a self-perceived instrument that evaluates the impact of dizziness and unsteadiness on the quality of life of patients with vestibular disorders [12,23]. The questionnaire consists of 25 items, divided into a seven-item physical subscale, a nine-item emotional subscale and a nine-item functional subscale. The total score ranges from 0 points (no handicap) to 100 points (severe handicap). To analyze DHI results, the total score, the categorical cutoff score >60 points, which indicates severe handicap [24], and a reduction in the score ≥18 points following treatment, which is considered significant improvement in the quality of life [25], will all be used.

The DGI is a functional gait scale composed of eight items with varying walking demands (ordinary walking, walking at different speeds, walking with vertical and horizontal head turns, walking over and around objects, making a 180° turn, and stair climbing) [26,27]. The total score ranges from 0 points (severe impairment) to 24 points (normal performance). To analyze DGI results, the total score, the categorical cutoff score <19 points for fall risk [28] and an increase in the score ≥4 points to identify a significant improvement following treatment [25] will be used.

Secondary outcome measures will include the following measures.

Dizziness features will be assessed using a visual analog scale, etiology (peripheral, central vestibular disorder, or both), time elapsed from the first occurrence, duration and frequency, and associated symptoms. Fall data will include the history of falls in the previous 6 months and fear of falling. Hand-grip strength (kg) will be assessed by the manual hydraulics dynamometer SAEHAN™ (SAEHAN Corporation, SH5001, Masan, South Korea).

The Activities-specific Balance Confidence Scale is a self-perceived measure of balance confidence in performing daily activities [29], with the score ranging from 0% (no confidence) to 100% (complete confidence).

The Vestibular Disorders Activities of Daily Living Scale evaluates the self-perceived impact of vestibular impairment on daily activities [30]. This scale consists of 28 questions divided into a twelve-item functional subscale, a nine-item ambulation subscale, and a seven-item instrumental subscale. The activities are classified according to a 10-point qualitative scale. The overall and subscales scores are calculated by taking the median values [31].

The Time Up and Go Test (TUG) is a mobility assessment tool [32], including versions with a dual task (TUG_cognitive_ and TUG_manual_) [33-35]. The test quantifies the time taken (seconds) to stand up, walk 3 m, turn, walk back, and sit down.

The Sit-to-Stand Test [36] is used to measure lower-extremity strength, postural control, and disability in older people and patients with vestibular disorders [37,38]. The measurement is obtained by the time required (seconds) to stand up and sit down five times, as quickly as possible.

The Multi Directional Functional Reach Test evaluates the limits of stability in the anterior–posterior and medial–lateral directions [39]. The measurement is obtained through the displacement reached by the patient (cm), shifting the center of gravity to the limits of the base of support while the feet remain stationary.

The static balance, the Romberg, the Romberg on unstable surface, the tandem position and the single leg stance tests [40,41] will all be tested for 30 seconds with eyes open and with eyes closed. The best time achieved over three trials is recorded.

The Geriatric Depression Scale is a 15-item screening questionnaire for symptoms of depression specifically for the older population [42,43]. The total score ranges from 0 to 15 points. A cutoff score ≥5 points indicates a depression mood [43].

Home exercise adherence will be assessed during and after the treatment, using a questionnaire developed by the researchers. Finally, adherence and adverse effects will also be recorded during the treatment sessions.

The following data will be collected at baseline to characterize the sample: social and demographic data (age, sex and education); anthropometric data (weight (kg), height (m), body mass index (kg/m^2^), upper limbs (cm), and feet length (cm)); health status (associated diseases, medication, use of assistive device (cane), complaint of musculoskeletal pain and smoking habit); and the Mini-Mental State Examination [21,44].

A follow-up questionnaire were developed by the researchers (satisfaction with treatment, hospitalization, unexpected clinical appointments, change in medication, use of medication for dizziness, new clinical diagnosis, occurrence of negative events in life) and will be also applied in order to detect possible problems that could interfere in the VR results in the long term.

The time frame and measures are depicted in Table 1. The assessments will be performed by trained physiotherapists. Each subject will be evaluated by the same outcome assessor at each time point.

**Table 1:** Measures and time frame

**Measure**	**Baseline**	**Post-treatment(8 weeks)**	**Follow**-**up(3 months)**
Primary outcome measures			
Dynamic Gait Index	✓	✓	✓
Dizziness Handicap Inventory	✓	✓	✓
Secondary outcome measures			
Dizziness features	✓	✓	✓
Fall data	✓	✓	✓
ABC scale	✓	✓	✓
VADL	✓	✓	✓
TUG (simple–manual– cognitive)	✓	✓	✓
Sit-to-stand test	✓	✓	✓
Multidirectional functional reach test	✓	✓	✓
Hand-grip strength	✓	✓	✓
Static balance	✓	✓	✓
Geriatric Depression Scale	✓	✓	✓
Home exercise compliance data		✓	✓
Adherence/satisfaction with treatment		✓	✓
Characterization data			
Social and demographic data	✓		
Health status	✓	✓	✓
Anthropometric data	✓		
Mini-Mental State Examination	✓		
Follow-up questionnaire			✓

ABC, Activities-specific Balance Confidence Scale; TUG, Time Up and Go Test; VADL, Vestibular Disorders Activities of Daily Living Scale.

### Sample size calculation

The estimated sample size was calculated considering previous DHI and DGI (primary outcomes) results based on a previously published trial [25], which compared VR outcomes in young versus older adults with vestibular disorders. The *t* test was used to detect clinical difference between means of continuous variants (primary outcomes) with 80% power, and 5% significance level. Estimations indicated that 34 individuals were required for the DGI (effect size = 2.5; standard deviation = 3.5) and 32 individuals for the DHI assessments (effect size = 14; standard deviation = 20), where effect size is the mean difference expected between the two groups and standard deviation is for the population. Hence, a minimum of 68 patients will be necessary for the sample. To minimize the effects of the dropout rate, at least 15% more individuals will be required to compose the final sample.

### Interventions

Interventions will be performed according to two distinct VR protocols (conventional Cawthorne&Cooksey and multimodal Cawthorne&Cooksey) conducted in the Otoneurology outpatient clinic. The VRs will be provided by two physiotherapists experienced in balance rehabilitation of older individuals and trained to standardize the VR protocols.

The control group will receive an active treatment, since scientific benefits of VR are already in evidence. In this case, placebo or nontreatment groups are not ethically advisable [45]. The control group subjects will thus be treated according to the conventional Cawthorne&Cooksey VR protocol [46-48], and the intervention group will be submitted to a novel protocol that includes multiple components to the conventional one.

The VR protocols will be provided in individual 50-minute sessions, twice a week, for 2 months (a total of 16 sessions). Under the condition of nonattendance, the session will be rescheduled to be performed in the same week. In the event of three (random or consecutive) absences during treatment, the subject will be excluded from the research and regarded as a withdrawal. In each session, absences, adverse symptoms, falling events, dizziness status according to the visual analog scale, and home exercises control will be recorded.

Regardless of the group, the subjects will be advised to maintain their usual activities and additional medical treatments during the research period. At the first session of both protocols, the subjects will receive a booklet including general information about VR, dietary advice, fall prevention and home exercises. The home exercises should be performed daily and consist of head and eye movements, performed while lying down (seven exercises, 14 minutes) and sitting (seven exercises, 14 minutes) to assure the patients’ safety. Following the recommendations, the intervention protocols will be implemented according to the randomization procedure.

Basically, the conventional Cawthorne&Cooksey protocol consists of eye, head and trunk exercises aimed at stabilizing the eye, reducing dizziness and improving body balance. The standard protocol comprises four stages, including specific exercises performed while lying down, sitting, standing, and walking positions. Each movement must be performed considering individual tolerability and avoiding neurovegetative symptoms. Lying and sitting exercises will take 1 week each; and, subsequently, standing and walking exercises will be undertaken during the following 6 weeks (3 weeks each) until the end of the intervention.

The Cawthorne&Cooksey modified protocol maintains the same exercises as the standard protocol, and includes flexibility, cognition, sensory interaction and muscle strength components. These alterations aim to match the various features of the ageing process, which is not restricted to vestibular problems alone, and to combine activities with higher functional demands.

**Figure 2:**
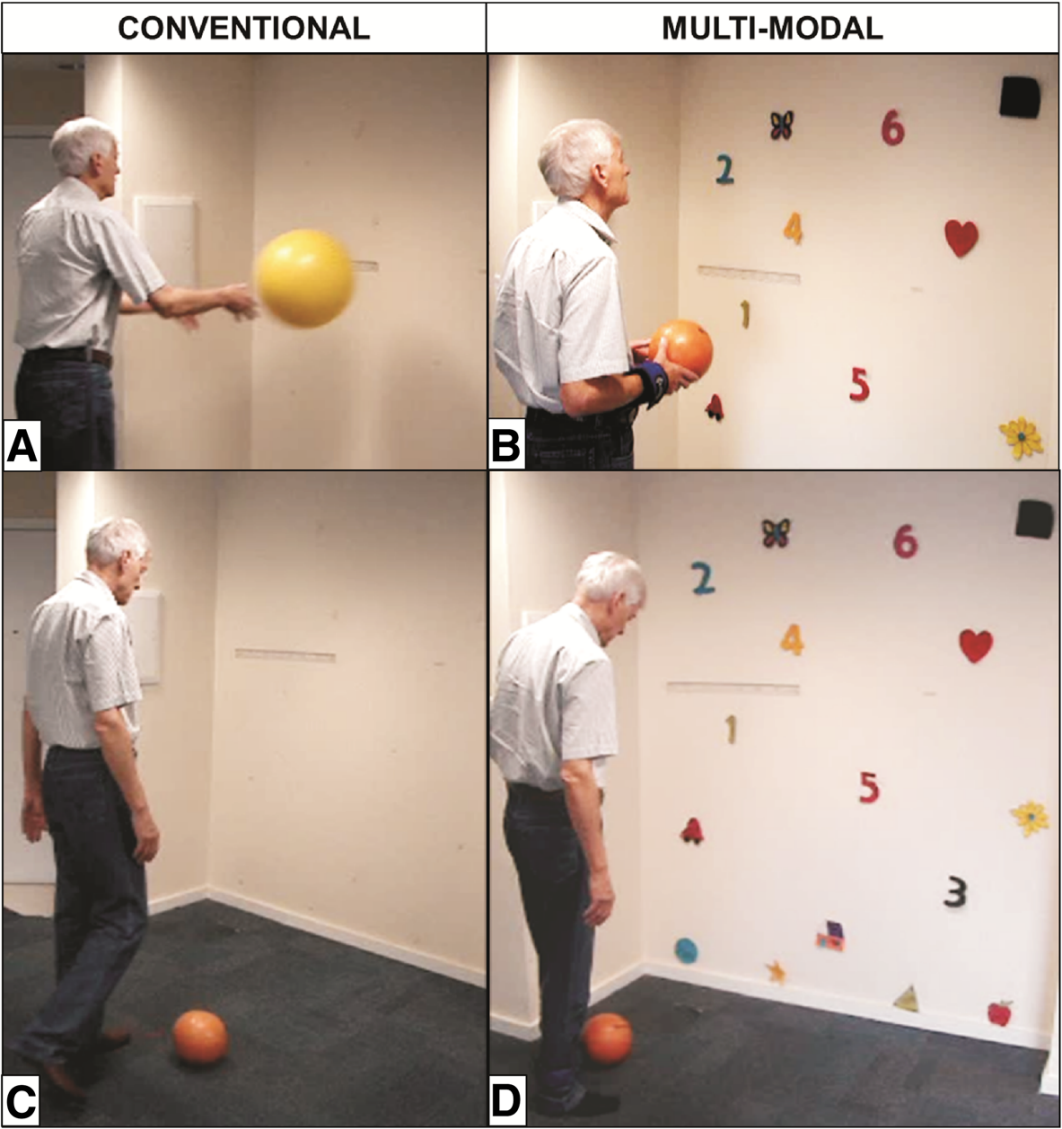
**Conventional and multimodal vestibular rehabilitation example of differences.** Stage D, Exercise A – throwing a ball: (**A**) conventional protocol and (**B**) multimodal protocol. Stage D, Exercise B – kicking a ball: (**C**) conventional protocol and (**D**) multimodal protocol.

The protocols are fully detailed in Additional file 1. Examples of the differences between both protocols are depicted in Figure 2.

### Statistical analysis

The effects of the VR protocols will be tested through both intention-to-treat and per-protocol analysis. For the intention-to-treat analysis, missing post-treatment or follow-up outcomes data will be replicated from previous measures available (assuming no change for noncompleters). For per-protocol analysis, data from excluded subjects will be disregarded for analysis. The groups will be compared at baseline by the chi-square test for qualitative data and by the *t* test for quantitative data. To analyze changes in outcomes at baseline, post-treatment, and follow-up between and within groups, analysis of variance with repeated measures will be applied. A normality test will be applied to the outcome measures; when data are not normally distributed, equivalent nonparametric tests will be used. Potential interactions between treatment and covariates, such as age, sex, comorbidities, medications, dizziness features, psychocognitive aspects, adherences and follow-up data, will also be tested. The results will be presented as frequencies/percentages for categorical variables, and as means, medians, standard deviations and 95% confidence intervals for continuous variables. Data analyses will be performed using SPSS for Windows version 17.0 (SPSS Inc., Chicago, IL, USA) and the significance value for all tests will be set at *P* <0.05.

## Discussion

The VR protocols are considered effective at reducing dizziness and its consequences [14,18]. However, there are few randomized clinical trials that investigate VR effects on exclusively older subjects, who, in addition to the vestibular disorder, present other impairments related to ageing that may negatively influence body balance control. Consideration of these conditions in addition to vestibular exercises might offer improve outcomes in this population. Since the Cawthorne&Cooksey VR protocol has already been recognized as effective, we believe that its modification with the inclusion of other components of postural control could improve its performance for older individuals with chronic dizziness. Both protocols are simple, require minimal resources and can be executed in various therapeutic settings, which enables their widespread use in case beneficial outcomes be observed. The comparison between rehabilitation protocols designed for older people with vestibular disorders would enable better planning and managing of interventions to diminish dizziness symptoms, functional disability and body imbalance, and to prevent falls in this population.

## Trial status

The study was concluded by September 2012. Outcome analysis and publish data will be performed on 2013.

## Abbreviations

DGI: Dynamic Gait Index; DHI: Dizziness Handicap Inventory; TUG: Time Up and Go Test; UNIFESP: Federal University of São Paulo; VR: vestibular rehabilitation.

## Competing interests

The authors declare that they have no competing interests.

## Authors’ contributions

NAR and MCA were responsible for the conception and design of the study. They also drafted the manuscript. HHC revised the manuscript critically for important intellectual content. FFG revised it critically for important intellectual content and made substantial contributions to the conception and design of the manuscript. All authors contributed to and take full responsibility for the paper and gave their final approval of the version to be published.

## Supplementary Material

Additional file 1 Document presenting the VR protocols (conventional versus multimodal). Click here for file
