# Supplementary material for: Effects of conventional versus multimodal vestibular rehabilitation on functional capacity and balance control in older people with chronic dizziness from vestibular disorders: design of a randomized clinical trial
Source: Trials. 2012 Dec 31;13:246. doi: 10.1186/1745-6215-13-246 (PMC3551791; doi:10.1186/1745-6215-13-246)
Supplement: Additional file 1 — Document presenting the VR protocols (conventional versus multimodal). [file 1745-6215-13-246-S1.doc]

| **Convencional** | | **Multi-modal** |
| --- | --- | --- |
| **Stage A- Booklet:** diet advice, falls prevention and home-based exercises | | |
| **Stage A- Lying Position- 28 minutes (1 session)** | | |
| 1. **Eye movements**   a) up and down.  b) from side to side.  c) focusing on finger moving from near to far away from face.   1. **Head movements**   a) bending forward and backward.  b) turning from side to side. | **1.** a-b-c:  2 min slow, 2 min quick.  **2.** a-b:  2 min slow, 2 min quick EO.  2 min slow, 2 min quick EC. | **1.** a-b-c:  2 min slow, 2 min quick.  Simultaneous counting backwards from a randomized number between 20 and 99.  **2.** a-b:  30 sec (2 series): neck stretching (flexor, extensor, rotator right, rotator left muscles).  1 min slow, 2 min quick EO.  1 min slow, 2 min quick EC. |
| **Stage B- Sitting Position- 32 minutes (2 sessions)** | | |
| **1. Eye movements**  Repeat a-b-c.  **2. Head movements**  Repeat a-b.  **3. Trunk movements**  a) shoulder shrugging and circling.  b) bending forward and picking up an object (medium ball) from the ground. | **1.** a-b-c:  2 min slow, 2 min quick.  **2.** a-b:  2 min slow, 2 min quick EO.  2 min slow, 2 min quick EC.  **3.** a:  2 min.  **3.** b:  2 min. | **1.** a-b-c:  2 min slow, 2 min quick.  Simultaneous fruit verbal fluency.  **2.** a-b:  30 sec (2 series): neck stretching.  1 min slow, 2 min quick EO.  1 min slow, 2 min quick EC.  **3.** a:  2 min: switching shoulder circling direction (counterclockwise- clockwise) every 30 sec.  **Session 1:** no wrist weight.  **Session 2:** 0.5 Kg wrist weight.  **3.** b:  30 sec (2 series) back stretching (sitting position- bending forward- holding).  1 min: switching the object every 30 sec.  **Session 1:** pencil/ medium ball.  **Session 2:** pencil/ heavy ball (2.0 kg). |
| **Stage C- Standing Position- 38 minutes (6 sessions)** | | |
| **1. Eye movements**  Repeat a-b-c.  **2. Head movements**  Repeat a-b.  **3. Trunk movements**  Repeat a.  **4. Dynamic movements**  a) sitting to standing position.  b) throwing a (small) ball from hand to hand above eye level.  c) throwing a (medium) ball from hand to hand under knees lifting foot.  d) sitting to standing position, and turning around oneself. | **1.** a-b-c:  2 min slow, 2 min quick.  **2.** a-b:  2 min slow, 2 min quick EO.  2 min slow, 2 min quick EC.  **3.** a:  2 min.  **4.** a-b-c-d:  2 min. | **1.** a-b-c:  4 min: switching feet position (2 min apart, 1 min feet together, 1 min tandem position).  **Sessions 1, 2:** stable surface.  **Sessions 3, 4:** unstable surface.  **Sessions 5, 6:** switching surface (stable- unstable) every 30 sec. Simultaneous counting backwards from number 20.  **2.** a-b:  4 min EO, 4 min EC: switching feet position (apart- together) at 2 min.  **Sessions 1, 2:** stable surface.  **Sessions 3, 4:** unstable surface.  **Sessions 5, 6:** switching surface (stable- unstable) every 30 sec. Simultaneous animal verbal fluency.  **3.** a:  2 min: switching shoulder circling direction (counterclockwise- clockwise) every 30 sec. Switching feet position/ surface (apart/ stable- together/stable- apart/unstable- together/ unstable) every 30 sec.  **Sessions 1, 2:** 0.5 Kg wrist weight.  **Sessions 3, 4:** 1.0 Kg wrist weight.  **Sessions 5, 6:** 1.5 Kg wrist weight.  **4.** a:  1 min regular seat height (43 cm), 30 sec higher seat height, 30 sec lower seat height.  **Session 1, 2:** higher (46 cm), lower (40 cm).  **Session 3, 4:** higher (49 cm), lower (37 cm).  **Session 5, 6:** higher (52 cm), lower (34 cm).  **4.** b:  2 min: switching ball size (little, small, medium, large) every 30 sec.  **4.** c:  2 min: switching ball size (little, small, medium, heavy- 2.0 Kg) every 30 sec.  **4.** d:  1 min EO, 1 min EC. |
| **Stage D- Moving- 24 minutes (6 sessions)** | | |
| **1.** Circling around the therapist who will throw a (medium) ball to you, and throwing it back.  **2.** Walking across the room.  **3.** Walking up and down the slope.  **4.** Walking up and down the steps.  **5.** Ball game  a) Throwing a (medium) ball.  b) Kicking a (large) ball. | **1.** 6 min.  **2**. 2 min EO, 2 min EC.  **3.** 2 min EO, 2 min EC.  **4.** 2 min EO, 2 min EC.  **5.** a:  3 min.  **5.** b:  3 min. | **1.** 6 min: switching ball size every 2 min.  **Sessions 1, 2:** little, medium, large balls.  **Sessions 3, 4:** small, medium, large balls.  **Sessions 5, 6:** small, large, heavy (2.0 Kg) balls. Simultaneous color verbal fluency.  **2.** 2 min EO, 2 min EC: switching walking direction (forward, backward, right, left) every 30 sec.  **Session 1:** no ankle weight.  **Session 2:** 0.5 Kg ankle weight.  **Sessions 3, 4:** 1.0 Kg ankle weight.  **Sessions 5, 6:** 1.5 Kg ankle weight.  **3**: 2 min EO: 1 min (holding a glass of water), 1 min (holding a ball).  **Session 1:** no ankle weight/ small ball.  **Session 2:** 0.5 Kg ankle weight/ medium ball.  **Sessions 3, 4:** 1.0 Kg ankle weight/ large ball.  **Sessions 5, 6:** 1.5 Kg ankle weight/ heavy (2.0 Kg) ball.  2 min EC  **Sessions:** Same ankle weight protocol (as above).  **4.**  2 min EO: 1 min regular step height (13 cm), 30 sec higher step height, 30 sec lower step height (6 cm).  **Session 1:** higher (16 cm)/ no ankle weight.  **Session 2:** higher (16 cm)/ 0.5 Kg ankle weight.  **Sessions 3, 4:** higher (19 cm)/ 1.0 Kg ankle weight.  **Sessions 5, 6:** higher (22 cm)/ 1.5 Kg ankle weight.  2 min EC: regular step height.  **Sessions:** Same ankle weight protocol (as above).  **5.** a: 3 min: throwing a ball towards targets attached to a wall according to the therapist’s instruction (2 min), and according to the patient’s own preference (1 min).  **Sessions 1-2:** small ball/ 0.5 Kg ankle weight.  **Sessions 3-4:** medium ball/ 1.0 Kg ankle weight.  **Sessions 5-6:** large ball/ 1.5 Kg ankle weight.  **5.** b: 3 min: kicking a ball towards targets attached to a wall according to therapist’s instruction (2 min), and according to the patient’s own preference (1 min).  **Sessions 1-2:** small ball/ 0.5 Kg wrist weight.  **Sessions 3-4:** medium ball/ 1.0 Kg wrist weight.  **Sessions 5-6:** large ball/ 1.5 Kg wrist weight. |
| min= minutes  sec= seconds  cm= centimeter  EO= Eyes Open  EC= Eyes Closed  Additional File 1: Vestibular Rehabilitation protocols (Conventional versus Multi-modal) | | |
